# Bearings Downsizing by Strength Enhancement and Service Life Extension

**DOI:** 10.3390/ma11091662

**Published:** 2018-09-08

**Authors:** Auezhan Amanov, Shirmendagva Darisuren, Young-Sik Pyun

**Affiliations:** Department of Mechanical Engineering, Sun Moon University, Asan 31460, Korea; shirmee9999@naver.com (S.D.); pyoun@sunmoon.ac.kr (Y.-S.P.)

**Keywords:** bearing, rolling contact fatigue strength, ultrasonic nanocrystalline surface modification (UNSM), hardness, roughness, super-polishing

## Abstract

Slim bearings are used widely in aircrafts, robots, wind turbines, and industrial machineries, where their size and weight are very important for the performance of a system. The common materials of slim bearings for robots and industrial machineries are based on SAE52110 bearing steel, and special heat treatment and a super polishing process are used and adapted to improve the rolling contact fatigue (RCF) strength of bearings. The improvement in RCF strength, depending on contact stress, surface hardness, and the friction behavior before and after ultrasonic nanocrystalline surface modification (UNSM) treatment was validated. Simple analysis shows that these improvements can reduce the size and weight of slim bearings down to about 3.40–21.25% and 14.3–26.05%, respectively. Hence, this UNSM technology is an opportunity to implement cost-saving and energy consuming super-polishing, a heat treatment process, and to reduce the size and weight of slim bearings.

## 1. Introduction

Todays’ requirements for extended service life and the increased downsizing of bearings are associated with reliability and the manufacturing cost of equipment. Slim, thrust ball, and spherical roller bearings are widely used in aircrafts, robots, wind turbines, and industrial machineries, where their size and weight are very important for the performance of a system. Rolling element bearings (REBs) are commonly used in tribo-machineries such as wind turbines, transmission, engines, etc. [1]. The size and weight of the inner and outer rings of slim bearings should be reduced as much as possible until they satisfy rolling contact fatigue (RCF) strength. The process of pitting failure involves the initiation of micro-cracks within the stressed volume via a damage accumulation process, subsequently followed by their growth, which eventually leads to the generation of surface pits and the ultimate failure of the component [2]. In this regard, common materials of the rings for aircraft jet engine bearings are special alloy steels, such as M series, and special heat treatment for refined micro grain, a high hardness of more than 63 HRC (Rockwell), compressive residual stress, and super finishing are usual processes for a very high cycle rolling strength of the rings. The general trend of an increase in fatigue life with increasing hardness has been observed earlier [3]. There are ceaseless efforts to develop new materials, a heat treatment process, and a super-polishing process to improve high cycle rolling contact fatigue strength, however their cost and energy are very high and even these technologies are controlled as confidential knowhow of suppliers and customers [4]. Materials in REBs are stressed during cyclic loading, resulting in the formation of irreversible subsurface microstructural alterations such as dark etching region (DER) and white etching bands (WEBs), slim bearings for robots, and a special reduction system of industrial machinery could not adapt such special alloy steel and processes due to high cost and confidential know-how. Consequently, general bearing steel such as SAE52100 is used for rings and rollers. DER has been reported to typically form under moderate to high contact stresses in the area of maximum shear stress after a high number of rolling cycles (5–100 × 10^6^ cycles). DER has also typically been found at a depth of approximately 0.10–0.65 mm below the contact surface [5]. DER typically spans between 0.5 and 2 mm in the depth direction, however it increases with running time and contact pressure.

Bearing life is influenced by the material microstructure, which is inherently inhomogeneous and, therefore, the fatigue life of an apparently identical batch of bearings operating under identical load, speed, lubrication, and environmental conditions will show a significant degree of scatter. Even a special heat treatment and polishing process have been developed for bearing materials, however their size and weight cannot be reduced due to the limit of RCF strength [6]. For example, Pramanic et al. have studied the fatigue life of machined components [7]. The super-polishing process reduced the surface roughness however, in turn, it removed the surface layers containing compressive residual stress, which tends to increase the fatigue strength of the polished components. Also, the super-polishing process may induce a tensile residual stress due to temperature rise. Moreover, the effects of bainitic-martensitic heat treatment on the microstructure and fatigue life of a bearing steel (SAE52100) have been investigated earlier [8]. It was found that no change in hardness was found, however the fatigue strength was enhanced by the shortened heat treatment process due to the homogenous dispersion of a small amount of austenite. The fatigue life of a bearing steel (SAE52100) by dynamic strain ageing has been investigated in a previous study [9]. It was reported that the dynamic strain ageing treatment introduced a more stable dislocation structure by increasing the mobile dislocation density and locking of these dislocations by diffusing carbon atoms and subsequently forming carbides. In addition to heat treatment, Kerscher and Lang have increased the fatigue life of a bearing steel (SAE52100) by deep cryogenic treatment [10]. Interestingly, the fatigue life increased only after some of the sequences. Hence, both the polishing and heat/cryogenic treatments sometimes may limit the fatigue life of bearings even though the processes increase the surface hardness, reducing the surface roughness, etc. In this regard, a surface modification technology, ultrasonic nanocrystal surface modification (UNSM), is a metal improvement technology which increases the mechanical properties and performance of materials [11]. The purpose of this study is to demonstrate a reduction in the size and weight of bearings for robots or special systems of industrial machineries by adapting a UNSM technology for the rings and rollers in order to reduce energy consumption and material cost. 

## 2. Validation of UNSM Technology 

The UNSM technology strikes the surface of a workpiece up to 20,000 times per second with a tungsten carbide (WC) and/or a silicon nitride (Si_3_N_4_) ball with a diameter range from 1.0 to 6.0 mm at a frequency of 20, 27, or 40 kHz that modifies the coarse grains into nano-sized grains until a certain depth from the top surface. The UNSM device includes an ultrasonic transducer, a horn, and an impacting tip, which comes into contact with the surface of a workpiece. The horn amplifies the high frequency ultrasonic vibrations that are generated by the transducer. Thus, the ball (tip) delivers static and dynamic loads to the workpiece. The most important advantage of the UNSM technology over other mechanical surface modification technologies is that the controllable static and dynamic loads provide a uniform and homogenous treatment. The main idea of UNSM technology is to introduce a high pressure of up to 30 GPa to a workpiece surface with high frequency strikes of more than 1 million times per minute and up to 100 K strikes per mm^2^, utilizing an ultrasonic energy and a resonance amplitude. These high dense and intense cycles pressure induces a high cycle of severe plastic deformation (SPD) up to a certain depth and high cycle of elastic deformation beneath this depth. The changes in mechanical properties and performance by UNSM treatment of a bearing alloy steel have been reported earlier [12]. In order to validate a possible reduction in the size and weight of slim bearings by adapting a UNSM technology, the increase in RCF strength needs to be examined using the experimental test specimens made of SAE52100 first, and a typical standard bearing can be selected to justify the effects in the same way on real standard bearings. The specimens and bearings were treated by UNSM technology under the parameters that are listed in Table 1. The reduction in the friction coefficient by reducing surface roughness and the formation of dimples via UNSM technology showed the possibility of eliminating or replacing a super-polishing process. Finally, the increased dynamic load rating of a typical slim bearings after UNSM technology will be derived based on the ISO standard of the dynamic load rating, and a possible reduction in size and weight of slim bearings will be analyzed and proposed.

### 2.1. RCF Strength of Ring Specimen by UNSM Technology

**Figure 1:**
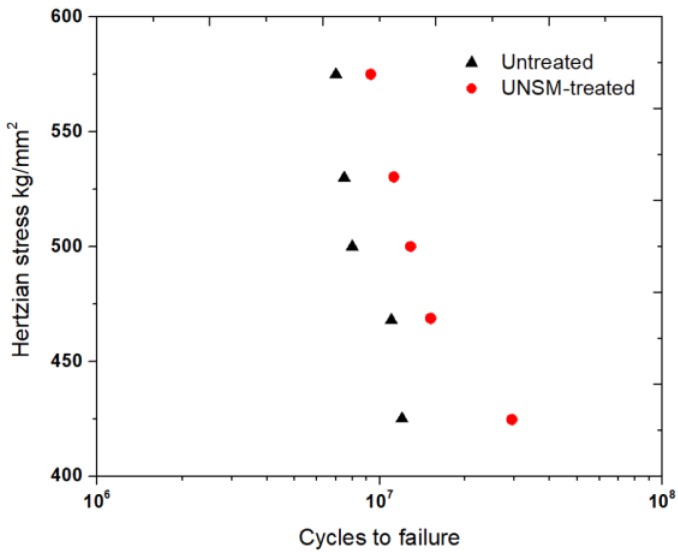
Comparison in S-N (stress-number of cycles) data of the untreated and UNSM-treated specimens at various contact stress levels.

Six-ball RCF test specimens with dimensions of 25 mm in diameter and 4 mm in thickness were used. Balls made of SAE52100 bearing steel with a diameter of 9.525 mm were used as rolling parts between the raceways. Six-ball RCF tests were conducted under elasto-hydrodynamic lubricating conditions at various contact stress levels, as shown in Table 2. When flaking occurred on the surface of the specimens, the vibration sensor detected it and the test was halted automatically. A comparison in cycles to flaking at various stress levels of the untreated and UNSM-treated specimens is shown in Figure 1. Thus, the compressive residual stress was induced from −1227 to −1343 MPa, with an increasing static load from 100 to 110 N, however it decreased from −1343 to −1091 MPa with an increasing static load of 110 to 120 N, as shown in Table 3. The compressive residual stress of the untreated may be induced by grinding and polishing processes during machining, and at a depth of about 40–80 µm, the compressive residual stress was eliminated. This indistinct phenomenon may be attributed to the microstructure modification that can be explained in terms of disordering grain dislocations and grain size refinement.

**Figure 2:**
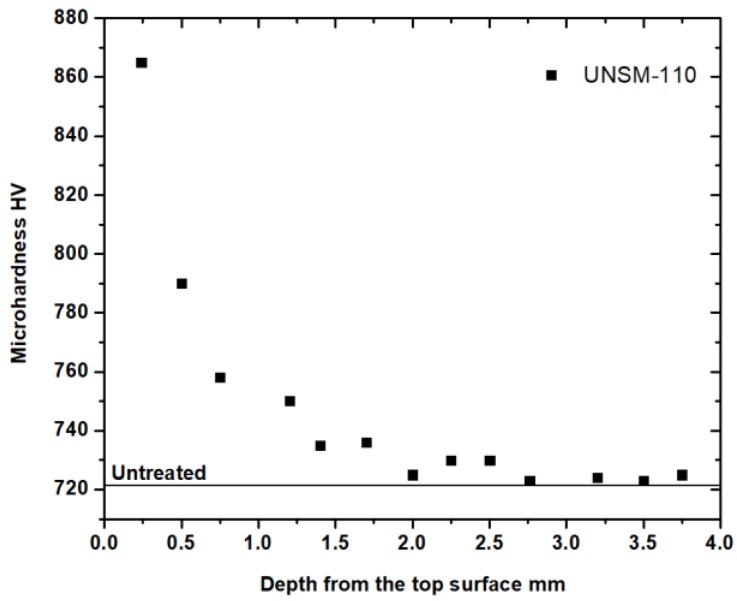
Variation of the micro-Vickers hardness value of the UNSM-110 specimens as a function of the depth from the top surface.

Figure 2 compares the micro-hardness of the untreated and UNSM-110-treated specimens, which was measured using a micro-Vickers hardness tester (MVK E3, Mitutoyo, Takatsui, Japan) at a load of 300 gf for a dwell time of 10 s. The top surface hardness of the specimen increased to up to 864 HV and then gradually decreased up to 720 HV, which is the hardness value of the untreated specimen. The hardness value of the UNSM-110 increased by about 20% compared to that of the untreated specimen. The increase in the hardness of the UNSM-110-treated specimen in comparison with the untreated one can be attributed to the grain size refinement by the Hall-Petch relationship, where the grain size plays an important role, and can also be attributed to work hardening effects [13].

**Figure 3:**
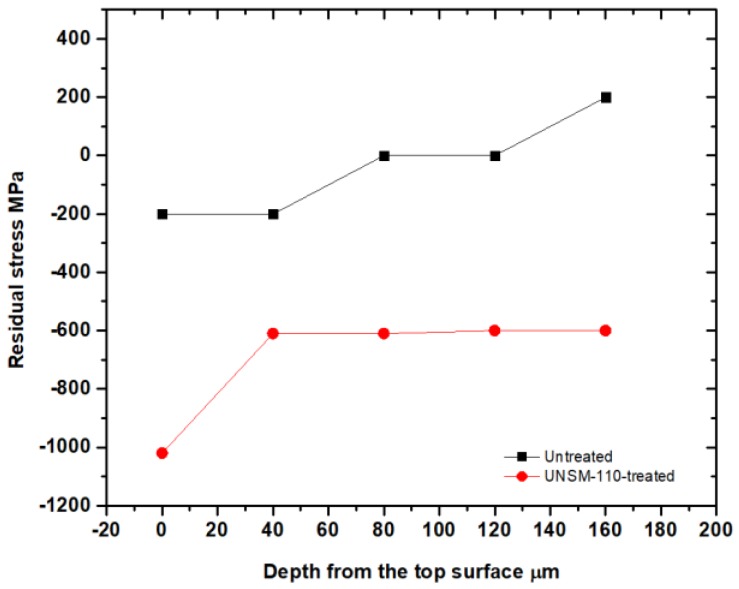
Variation of the compressive residual stress value of the untreated and UNSM-110-treated specimens as a function of the depth from the top surface.

The residual stress results of the untreated and UNSM-110-treated specimens are shown in Figure 3. It shows that the compressive residual stress value from the top surface to the center of the specimen gradually decreased and saturated the residual value in the case of the UNSM-treated specimen, however it rapidly decreased in the untreated specimen. It is interesting to note that the compressive residual stress was not linearly reduced with the increasing impact load of the UNSM technology. The induced compressive residual stress by the UNSM technology can be explained due to the grain size refinement through SPD and stress relieving [14], and it is the major factor in increasing the fatigue strength and crack growth rate of bearings [15,16]. Also, it has been reported earlier that the UNSM technology is able to transfer a tensile residual stress into a compressive residual stress [17]. In addition, the degree of the residual stress depends on the UNSM parameters, however there is a need to confirm the values and distribution with respect to depth. However, there is a need to confirm their values and distribution through the depth induced compressive residual stress which was −1227 to −1343 MPa with an increasing static load from 100 to 110 N, respectively, however it decreased from −1343 to −1091 MPa with an increasing static load of 110 to 120 N, respectively, as shown in Table 3. This indistinct phenomenon may be attributed to the microstructure modification that can be explained in terms of disordering grain dislocations, dislocation formation and grain growth. However, it is worth mentioning here that there is a limitation on grain size refinement by SPD methods, where a refined grain size that is less than 10 nm may deteriorate the mechanical and other properties of materials [18].

### 2.2. Validation in Thrust Ball Bearing Fatigue Life Test 

In order to justify the effect of UNSM technology in real bearings, a thrust ball bearing was selected and a comparison test was performed at a Hertzian stress of 5 GPa with 1500 rpm under oil lubricated conditions. At least three samples were used for each fatigue result due to data scattering. Average cycles to failure of the untreated new bearing was found to be 1.81 × 10^6^ cycles and the UNSM-treated bearing was run-out after 3.06 × 10^6^ cycles, as shown in Table 4.

### 2.3. RCF Strength of Rollers by UNSM Technology

The RCF test specimens made of SAE52100 with a diameter of 15 mm and a length of 300 mm were used. The result of the effect of repeating load being applied to the specimen on the RCF life showed that the fatigue life was shortest at 122 N and it increased more than 3 times from 13 × 10^6^ to 41 × 10^6^ cycles after UNSM treatment, even under the same load of 1200 N and with a rotation speed of 8000 rpm. SPD that was caused by repeated maximum shear stress during RCF test increased dislocation density and the stress-induced diffusion of C atoms, which are supersaturated in surrounding martensite which occurs along the high diffusion paths, which results in the formation of deformation bands along maximum shear.

### 2.4. Validation in Spherical Roller Bearing Fatigue Life Test

In order to justify the effect of UNSM technology in real bearings, a spherical roller bearing (designation FAG24020) was selected for a comparison test that was performed under the test conditions that are listed in Table 5. Cycles to failure under the Herzian stress of 2.9 GPa of the untreated and UNSM-treated bearings was 6.5 × 10^5^ and 1 × 10^6^ run out, as listed in Table 6. 

### 2.5. Validation in Fatigue Test of Bearing Ring 

In order to justify the effect of UNSM technology in real bearings, a small ring of taper roller bearing (30210A) was selected for comparison tests. At a maximum stress of 1.29 GPa, the untreated ring was failed at 8 × 10^5^ cycles, while the UNSM-treated ring was runout after 10 × 10^6^ cycles. 

### 2.6. Possibility to Eliminate Super Polishing Process of Ring and Roller 

**Figure 4:**
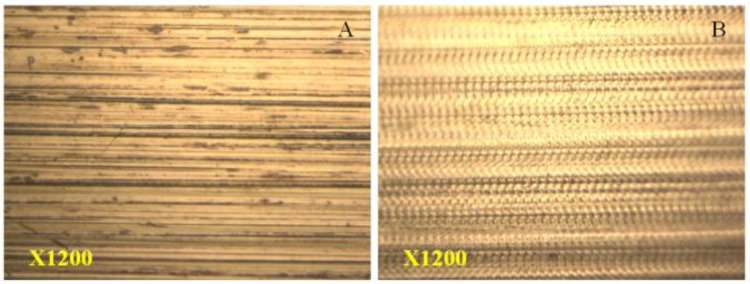
Polished (**A**) and UNSM-treated (**B**) roller surface of the taper roller bearings.

The main purpose of super-polishing the raceways is to increase λ factor under the lubrication regimes. This is defined as oil film thickness divided by equivalent surface roughness of both of the mating surfaces and to reduce the friction coefficient. The average surface roughness (Ra) of the normal thrust ring of 0.18 µm reduced to 0.08 µm, where the surface structure was changed into a dimpled/textured structure, as shown Figure 4. The exact dimensions, such as the diameter and depth of the produced dimples can be found in the previous study [11]. The surface roughness of the untreated roller with a diameter of 43 mm was about 0.35 µm, which reduced to 0.21 µm after UNSM technology. The dimpled surface that is shown in Figure 4B may be produced on the surface of the bearing raceway to reduce the friction coefficient and wear rate during the rolling and sliding in the interaction contact of the ball and the raceway. The deformation-induced transformation mechanism can explain the surface hardening through the compressive residual stress and increase in the dislocation density. The average friction coefficient at the variation with rotation speed and load can be reduced by 22 ~ 39% [14]. Hence, except for aircraft bearings, the super polishing of slim ring bearings for the surface roughness *(*Ra*)* of 0.06 µm could be replaced by 0.08 µm and micro dimples by UNSM technology.

## 3. Possibility of Downsizing of Slim Bearings

The dynamic load rating of bearings is the main factor to select a proper specification of bearings and could be derived by ISO standard as Equations (1) and (2) [19] for ball and roller bearings, respectively. The L_10_ service life of bearings could also be derived by those equations. When it is supposed that the improved RCF has the same effect on the L_10_ condition, the increased basic dynamic load rating could be derived from Equations (5) and (6) [19] for the ball and roller bearings, respectively. A summary of RCF for the ball and roller test results that were obtained at a contact stress of 4.2 GPa are listed in Table 7 and Table 8.

For ball bearings:

(1) $$L_{10}={\left(\frac{C}{P}\right)}^{3}$$

For roller bearings:

(2) L10=(CP)103

where: L_10_ is the fatigue life that 90% of a sufficiently large group of apparently identical bearings can be expected to reach or exceed; P is the equivalent dynamic bearing load; C is the basic dynamic load rating of the ball bearings that were obtained from Equation (1) as follows:

L1013=CP→C=L1013·P

It is possible to design the improved range of dynamic load. The minimum value for ring, RCF (ball) could be as follows:

CUNSM=L1013·P=1.2966·P

It is possible to design the improved range of dynamic. The maximum value for the ball bearing fatigue test could be as follows:

CUNSM=L1013·P=6.96·P

The UNSM-treated ball bearings C_UNSM_ could be derived as Equation (3):

(3) $${1.29\;C<C}_{\mathrm{UNSM}}<1.9$$

C was obtained from Equation (2) as follows:

L10310=CP→C=L10310·P 

It is possible to design the improved range of dynamic. The maximum value for the RCF (roller) fatigue test could be as follows:

CUNSM=L10310·P=3.15310·P=1.41·P

It is possible to design the improved range of dynamic. The maximum value for the roller fatigue test could be as follows:

CUNSM=L10310·P=1.55310·P=1.14·P

The UNSM-treated roller bearings C_UNSM_ could be derived as Equation (4):

(4) $${1.14\;C<C}_{\mathrm{UNSM}}<1.41$$

### 3.1. Downsizing of Slim Ball Bearing

A typical slim ball bearing with an outer diameter of 177.8 mm and an inner diameter of 165.0 mm and a width of 6.35 mm was used. Table 9 shows the possibility of reducing the size and weight of a bearing by UNSM technology.

The basic dynamic load rating Equation (5) [19] for radial ball bearings.

(5) C=fcm(icosα)0.7Z23D1.8

UNSM-A C_UNSM_

$$\gamma =\frac{\mathrm{Dcos}\alpha }{d_{m}}=\frac{3\cos30}{170}=0.0152$$

C=fcm(icosα)0.7Z23D1.8=5589.2 N

$$C_{\mathrm{UNSM}}=C\cdot 1.297=7249.2\;N$$

UNSM-B C_UNSM_

$$\gamma =\frac{\mathrm{Dcos}\alpha }{d_{m}}=\frac{3\cos30}{166}=0.0156$$

C=fcm(icosα)0.7Z23D1.8=5589.2 N

$$C_{\mathrm{UNSM}}=C\cdot 1.910=10,675.3\;N$$

where: f_cm_ is the factor for calculating C; i is the number of rows of balls; α is the contact angle, degree; z is the number of rolling elements per row; D is the diameter of the balls.

**Figure 5:**
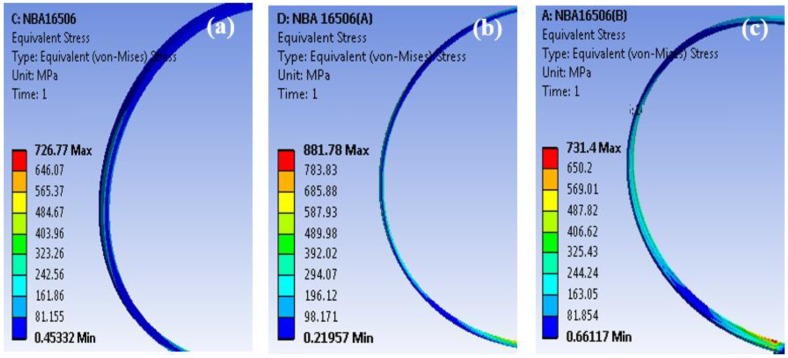
ANSYS equivalent stress result of angular contact ball bearings of outer ring: (**a**) untreated ring, (**b**) UNSM-A ring, (**c**) UNSM-B ring.

In the case where the inner diameter is kept the same, the ball size could be reduced from 3.9 to 3 mm and the on and width could be reduced to 6.35 and 6 mm, while the dynamic load rating with UNSM technology maintains the same level. The weight could be reduced from 0.119 to 0.088 kg. In the case of the inner diameter, it could also be changed from 165 to 160 mm, and the ball size could be reduced from 3.9 to 3 mm also. So, the outer diameter and width could be reduced to 6.35 and 5 mm, while the dynamic load rating with UNSM technology could be maintained at the same level. The weight could be reduced from 0.119 to 0.085 kg. The maximum equivalent stress that is induced by normal force at the outer ring are analyzed by finite element analysis (FEA) and are compared, as shown in Figure 5 and Table 10. When considering the increased fatigue strength by 28% after UNSM technology on the ring, the fatigue life of the reduced size of the bearings needs to be longer than the untreated bearings.

### 3.2. Downsizing of Slim Roller Bearing

The dimensions of a typical slim roller bearing with an outer diameter of 150 mm, an inner diameter of 100 mm, and a width of 24 mm are listed in Table 11, which shows the possibility of reducing the size and weight of a bearing by UNSM technology.

The basic dynamic load rating Equation (6) [19] for radial roller bearings:

(6) C=fcm(iLcosα)79Z34D2927

UNSM-A C_UNSM_

$$\gamma =\frac{\mathrm{Dcos}\alpha }{d_{m}}=\frac{11\cos0}{125.5}=0.087$$

C=fcm(iLcosα)79Z34D2927=4524.8 N

$$C_{\mathrm{UNSM}}=1.41\;C=6379.9\;N$$

UNSM-B C_UNSM_

$$\gamma =\frac{\mathrm{Dcos}\alpha }{d_{m}}=\frac{11\cos0}{120}=0.091$$

C=fcm(iLcosα)79Z34D2927=4524.8 N

$$C_{\mathrm{UNSM}}=1.41\;C=5158.3\;N$$

**Figure 6:**
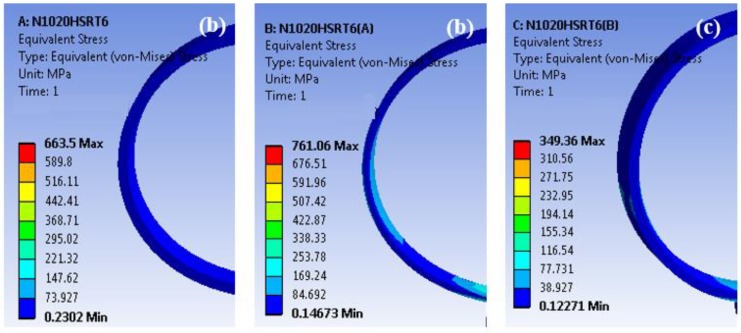
ANSYS equivalent stress result of roller bearings of the outer ring: (**a**) untreated ring, (**b**) UNSM-A ring, (**c**) UNSM-B ring.

The maximum equivalent stress induced by normal force at the outer ring are analyzed by FEA (ANSYS Inc., Canonsburg, PA, USA) and are compared, as shown in Figure 6. Considering the increased fatigue strength by 28% after UNSM treatment on the ring is shown in Table 12. 

In cases where the inner diameter is kept the same, the roller size could be reduced from 12 × 10 to 11 × 9 mm^2^ and the outer diameter and width could be reduced to 24 and 20 mm, while the dynamic load rating with UNSM technology is maintained at the same level. The weight could be reduced from 1.05 to 0.74 kg. In cases when the inner diameter could be reduced from 100 to 95 mm, the roller size could be reduced from 12 × 10 to 10 × 9 mm^2^ as well. So, the outer diameter and width could be reduced to 24 and 20 mm^2^, while the dynamic load rating with UNSM technology is maintained at the same level. The weight could be reduced from 1.05 to 0.90 kg. The maximum equivalent stress on both rings of three bearings under dynamic load rating and static load rating are compared in Table 12. These results can validate that the reduced size of the bearings will have a longer service life in RCF than those of the original bearings, respectively. The possibility of reducing the size and weight of slim bearings is also summarized in Table 9 and Table 11.

## 4. Conclusions

In this study, the fatigue results and improvement percentages of the untreated and UNSM-treated specimens were obtained within the performed experimental metrics. This study validated that the RCF strength could be improved by about 118 and 28%, respectively, while cycles to failure on RCF of thrust ball and spherical roller bearings could be increased by about 596% and 55%, respectively. Also, the friction coefficient of thrust ball bearings could be reduced by about 55 ~ 118% by UNSM treatment depending on treatment parameters. The analysis showed that the weight of slim ball and roller bearings could be reduced by about 3.40 ~ 21.25% and 14.3 ~ 26.05%, respectively. Hence, a huge amount of materials and energy can be saved by reducing the weight and size of bearings by adapting a UNSM technology.

## Figures and Tables

**Table 1:** UNSM treatment parameters.

Frequency kHz	Amplitude µm	Horn Speed mm/min	Feed-Rate mm/rev	Rotating Speed rpm	Load N	Ball Diameter mm
20	30	3000	0.07	115	60	2.38

**Table 2:** RCF test conditions.

Hertzian Contact Stress MPa	Rotating Speed rpm	Lubricant
4250	1000	Shell Tellus 37
4750		
5000		
5300		
5800		

**Table 3:** The mechanical properties of the untreated and UNSM-treated specimens.

Static Load of the UNSM Treatment	Surface Hardness HV	Surface Roughness (R_a_) µm	Residual Stress MPa
Untreated	720	0.344	−200 (top surface) 200 at a depth of 160 µm
UNSM-100	850	0.110	−1227
UNSM-110	864	0.105	−1343
UNSM-120	855	0.115	−1091

**Table 4:** RCF test results.

New Bearing	Used Bearing	Used Bearing Treated by UNSM
1.81 × 10^6^ cycles	4 × 10^5^ cycles	3 × 10^6^ cycles

**Table 5:** Spherical roller bearing fatigue life test conditions.

Time, h	Load, kN	Rotating Speed, rpm	Lubrication Type
83.24	255	200	ISO VG 46

**Table 6:** Spherical roller bearing fatigue life test results.

Spherical Roller Bearing	Time h	Rotating Speed rpm	Ratio %
Untreated	53.47	6.5 × 10^5^ run out	100.0
UNSM-treated	83.24	1 × 10^6^ run out	155.0

**Table 7:** Summary of fatigue test results.

Fatigue Test	Treatment	Fatigue, Cycles	Ratio, %
RCF (ball)	Untreated	4.641 × 10^6^	100.0
	UNSM-treated	10.119 × 10^6^	218.0
RCF (Roller)	Untreated	12.96 × 10^6^	100.0
	UNSM-treated	40.88 × 10^6^	315.0
Ball bearing fatigue test	Untreated	0.4395 × 10^6^	100.0
	UNSM-treated	3.06 × 10^6^	696.0
Roller bearing fatigue test	Untreated	6.454 × 10^5^	100.0
	UNSM-treated	1 × 10^6^ run out	155.0

**Table 8:** Basic dynamic load rating of the UNSM-treated specimens.

Fatigue Test	Treatment	C-Basic Dynamic Load Rating of Bearings
RCF (ball)	Untreated	Equation (1)
	UNSM-treated	1.297 × P
RCF (roller)	Untreated	Equation (2)
	UNSM-treated	1.41 × P
Ball bearing fatigue test	Untreated	Equation (1)
	UNSM-treated	1.910 × P
Roller bearing fatigue test	Untreated	Equation (2)
	UNSM-treated	1.14 × P

**Table 9:** The possibility of reducing the size and weight of a slim ball bearing by UNSM technology at two different dynamic load ratings UNSM A and UNSM B.

Dimensions	Meanings	Untreated	UNSM A	UNSM B
d, mm	bearing inner diameter	165.0	165.0	160
D, mm	bearing outer diameter	177.8	175.3	172
B, mm	bearing width	6.35	6	5
L, N	dynamic load	C	C_1.297-UNSM_	C_1.14-UNSM_
		-	7249.2	10,675.3
d_ball_, mm	ball diameter	3.9	3	3
		57	66	66
W, kg	bearing weight	0.119	0.088	0.85

**Table 10:** Comparison in stress results of angular contact ball bearings for the untreated and two different dynamic load ratings UNSM A and UNSM B.

Bearings	Load N	Stress MPa	UNSM 28% (Increase in Fatigue Strength by UNSM Technology by 28%)
Untreated	100	726.8	-
UNSM A	100	881.9	1128.8
UNSM B	100	731.3	936.0

**Table 11:** The possibility of reducing the size and weight of slim roller bearings by UNSM technology at two different dynamic load ratings UNSM A and UNSM B.

Dimensions	Meanings	Untreated	UNSM A	UNSM B
d, mm	bearing inner diameter	100	100	95
D, mm	bearing outer diameter	150	145	140
B, mm	bearing width	24	20	20
Load, N	dynamic load	C	C_1.41-UNSM_	C_1.14-UNSM_
		-	6379.9	5158.3
d_ball_, mm	ball diameter	12	11	11
		10	9	9
		12	13	13
W, kg	bearing weight	1.05	0.74	0.90

**Table 12:** Comparison in stress results of roller bearings for the untreated and two different dynamic load ratings UNSM A and UNSM B.

Bearings	Load N	Stress MPa	UNSM 28% (Increase in Fatigue Strength by UNSM Technology by 28%)
Untreated	500	663.5	-
UNSM A	500	761.0	974.1
UNSM B	500	349.4	447.2
